# Study protocol: the DESPATCH study: Delivering stroke prevention for patients with atrial fibrillation - a cluster randomised controlled trial in primary healthcare

**DOI:** 10.1186/1748-5908-6-48

**Published:** 2011-05-20

**Authors:** Melina Gattellari, Dominic Y Leung, Obioha C Ukoumunne, Nicholas Zwar, Jeremy Grimshaw, John M Worthington

**Affiliations:** 1 School of Public Health and Community Medicine, The University of New South Wales, Sydney, Australia; 2 Department of Cardiology, Liverpool Health Service, South Western Sydney Local Health Network, Liverpool, Australia; 3 South Western Sydney Clinical School, The University of New South Wales, Sydney, Australia; 4 PenCLAHRC, Peninsula College of Medicine and Dentistry, University of Exeter, Exeter, UK; 5 Ottawa Health Research Institute, Ottawa, Ontario, Canada; 6 Department of Neurophysiology, Liverpool Health Service, South Western Sydney Local Health Network, Liverpool, Australia; 7 Northern Beaches Hospitals, Northern Sydney Local Health Network, Manly and Mona Vale Hospitals, Manly, Australia

## Abstract

**Background:**

Compelling evidence shows that appropriate use of anticoagulation in patients with nonvalvular atrial fibrillation reduces the risk of ischaemic stroke by 67% and all-cause mortality by 26%. Despite this evidence, anticoagulation is substantially underused, resulting in avoidable fatal and disabling strokes.

**Methods:**

DESPATCH is a cluster randomised controlled trial with concealed allocation and blinded outcome assessment designed to evaluate a multifaceted and tailored implementation strategy for improving the uptake of anticoagulation in primary care. We have recruited general practices in South Western Sydney, Australia, and randomly allocated practices to receive the DESPATCH intervention or evidence-based guidelines (control). The intervention comprises specialist decisional support via written feedback about patient-specific cases, three academic detailing sessions (delivered via telephone), practice resources, and evidence-based information. Data for outcome assessment will be obtained from a blinded, independent medical record audit. Our primary endpoint is the proportion of nonvalvular atrial fibrillation patients, over 65 years of age, receiving oral anticoagulation at any time during the 12-month posttest period.

**Discussion:**

Successful translation of evidence into clinical practice can reduce avoidable stroke, death, and disability due to nonvalvular atrial fibrillation. If successful, DESPATCH will inform public policy, providing quality evidence for an effective implementation strategy to improve management of nonvalvular atrial fibrillation, to close an important evidence-practice gap.

**Trial registration:**

Australia and New Zealand Clinical Trials Register (ANZCTR): ACTRN12608000074392

## Background

### An evidence-practice gap

Nonvalvular atrial fibrillation (NVAF) is a common arrhythmia of the heart that increases the likelihood of stroke and transient ischaemic attack (TIA), through clot embolism to large arteries of the brain [1]. NVAF is more prevalent with increasing age, affecting 1 in 20 people over the age of 65 and 1 in 10 over 75 [2]. Overall, NVAF accounts for 15% of stroke cases but as many as 20% of strokes in those aged 70 to 79 years and 30% of strokes in people aged 80 to 89 years [2,3]. The risk of stroke associated with NVAF depends on the presence of other stroke risk factors. A commonly used algorithm, called the CHADS2 score (congestive heart failure (CHF), hypertension, age over 75 years, diabetes and either prior stroke or TIA) [4], has been recommended to calculate the stroke risk in NVAF [5]. One point each is assigned for the presence of CHF, hypertension, age over 75 years and diabetes and two points for either prior stroke or TIA. Predicted annual stroke risk varies from 1.9% for a CHADS2 score of 0 to 18.2% for a score of 6.

Over 20 years of evidence from several randomised controlled trials demonstrates the effectiveness of antithrombotics in reducing the risk of ischaemic stroke in NVAF [6,7]. Antithrombotic agents are classified as either anticoagulants (*e.g*., warfarin) or antiplatelets (*e.g*., aspirin or clopidogrel). Anticoagulation dosing with warfarin is usually adjusted (adjusted-dose warfarin), according to blood tests, to maximise the benefits of treatment and minimise bleeding risk. Compared with placebo or no treatment, adjusted-dose warfarin reduces the risk of ischaemic stroke in patients with NVAF by 67% in relative terms (95% confidence interval [CI], 54% to 77%) [6]. Warfarin also reduces all-cause mortality by 26% (95% CI, 3% to 43%) [6]. Aspirin, the most widely studied antiplatelet medication, is associated with a more modest relative risk reduction (RRR) for ischaemic stroke (21%; 95% CI, -1% to 38%) [6]. Head-to-head comparisons of stroke risk reduction favour adjusted-dose warfarin over aspirin (RRR = 52%; 95% CI, 41% to 62%) and newer antiplatelet treatments [6-8].

Until recently, the management of NVAF in the elderly (> 80 years) remained problematic. Existing trials had typically enrolled younger patients (average age 71 years), perceived as less vulnerable to the risks of iatrogenic haemorrhage on warfarin [6]. There was also uncertainty about whether the benefits of warfarin could be realised in the 'real-world' setting of primary healthcare, compared with trial settings in tertiary institutions.

The Birmingham Atrial Fibrillation Treatment of the Aged (BAFTA) trial demonstrated the benefits of warfarin in primary healthcare in the elderly, randomising patients with an average age of 81.5 years to receive either warfarin or aspirin [8]. Patients were recruited into the study by their primary healthcare physicians, who were also responsible for patient day-to-day management. After an average of 2.7 years of follow-up, results showed that warfarin reduced the risk of ischaemic stroke by 70% (95% CI, 37% to 87%) and the risk of any major vascular event, including any stroke, myocardial infarction, pulmonary embolus, and vascular death, by 27% (95% CI, 1% to 47%). The risk of any major haemorrhage, including haemorrhagic stroke, was similar between patients receiving warfarin or aspirin (1.9% per year vs. 2.0%). The BAFTA study confirmed that warfarin is more effective than aspirin in the elderly receiving routine care and can be as safe as aspirin in older patients managed in a primary healthcare setting.

### Previous studies and other work informing this trial

Despite this evidence, recent reports suggest that up to 50% of patients with NVAF are not prescribed anticoagulation [*e.g*., [9]. At the time of planning this study, no single intervention had been shown to improve the management of NVAF in primary healthcare and the uptake of appropriate antithrombotics when evaluated in a randomised controlled trial. In a randomised evaluation of a patient decision aid, McAlister *et al*. [10] reported an increase in antithrombotic prescribing at three months following the intervention. However, at 12 months, the rates of antithrombotic prescribing in the intervention group had reverted to baseline levels and did not differ from the control group. Ornstein *et al*. [11], in a multifaceted intervention targeting several cardiovascular risk factors in primary healthcare, including atrial fibrillation, evaluated the effect of audit and feedback and computerised guidelines and reminder systems for overcoming practical and organisational barriers. Anticoagulant prescribing decreased over time in the intervention group, and no significant differences in prescribing were observed at posttest between intervention and control groups. In a trial carried out in general practices in England, practices were randomised to receive locally adapted guidelines, one educational meeting delivered by local opinion leaders, educational materials, and an offer of one educational outreach visit (or academic detailing) to improve the management of TIA and atrial fibrillation [12]. This intervention did not increase compliance with antithrombotic prescribing guidelines; however, the outcome did not distinguish between prescribing for warfarin or aspirin. A nonrandomised study, carried out in Tasmania, Australia, demonstrated a promising effect of guideline dissemination followed by academic detailing visits to primary healthcare physicians in one region in Tasmania [13]. The prescribing and use of warfarin had significantly increased within the intervention region but not the control region. However, as this study did not employ a randomised design, it is unclear, whether or not this result was biased by confounding variables.

Studies suggest that strategies to improve the management of NVAF should address physicians' concerns about the risks of anticoagulation. Choudhry *et al*. [14] reported that physicians were less likely to prescribe warfarin for patients with NVAF after any one of their patients receiving warfarin was admitted to a hospital for a haemorrhage. Physicians were no more or less likely to prescribe warfarin, however, if any one of their patients with NVAF had been admitted to a hospital with an ischaemic stroke.

In our representative survey of 596 Australian primarycare physicians, known in Australia as general practitioners (GPs), the GPs appeared overly cautious in prescribing anticoagulation in the presence of any perceived risk of major and even minor bleeding, even where treatment benefits clearly outweighed the risk of harm [15,16]. A substantial proportion of GPs 'strongly agreed' or 'agreed' that they were 'often unsure whether or not to prescribe warfarin' and that 'it is hard to decide whether the benefits of warfarin outweigh the risks or vice versa' (30.0% and 38.4%, respectively). Other local surveys have indicated GP reluctance to prescribe anticoagulation for NVAF in the elderly or in the presence of perceived bleeding risks, which would not necessarily preclude anticoagulation on the available evidence [17,18].

Clinicians with perceived specialist knowledge in stroke prevention and atrial fibrillation may be effective educators and preceptors for improving clinical management of NVAF. Yet, such access to experts in stroke medicine seems limited. In our national survey, a significant proportion of Australian GPs were either 'dissatisfied' or 'very dissatisfied' with access to neurologists (51.8%), even in metropolitan settings (47.4%) (Gattellari, Zwar, Worthington, unpublished data). Previous research has found that collaborative involvement of specialists with family physicians increases anticoagulation prescribing in patients, suggesting collaboration with specialists is an important factor in patient care [19].

We set out to develop and evaluate a multifaceted, educational intervention (DESPATCH) tailored to the self-identified needs of Australian GPs, recognising their high perceived risk of anticoagulant use and the likely value of building confidence in decision making. The intervention features peer academic detailing and educational and practice materials. A novel element is expert decisional support to promote the uptake of anticoagulation, using feedback from clinical experts in stroke medicine.

Our primary hypothesis is that a higher proportion of patients with NVAF whose GPs have been randomly allocated to receive the DESPATCH intervention will be prescribed oral anticoagulation medication compared with patients whose GPs are allocated to the control group.

## Methods

### GP recruitment

All GPs located in our local region, South Western Sydney, were selected from a commercial database containing the contact details of GPs in active practice [20]. We restricted the population to GPs practicing with up to five other GPs to avoid large medical centres where GPs, practice staff, and patients are more likely to be itinerant. GPs were located within postal codes of the geographically defined regions, known as Local Government Areas (LGAs), of Fairfield (population 190,657), Campbelltown (population 149,071), Camden (population 53394), Bankstown (population 182,178), Liverpool (population 176,903), Canterbury (population 139,985), and Marrickville (population 77,141) [21]. GPs were mailed a prenotification letter advising them that researchers from the Faculty of Medicine of the University of New South Wales were offering the opportunity to participate in an education program about stroke prevention in general practice. The letter advised GPs that a research nurse would phone their practice to arrange a practice visit to explain the study in detail and obtain written consent. This professional development program was accredited by the peak professional body representing GPs in Australia (The Royal Australian College of General Practitioners).

### Inclusion criteria

GPs were eligible only if their practice utilised an electronic register recording contact details for patients, their date of birth, and date of last consultation. GPs were required to use their electronic system for recording prescriptions to facilitate identification of patients with NVAF.

### Exclusion criteria

GPs who anticipated retiring or moving their practice within the next 12 months were ineligible to participate.

### GP questionnaire

Prior to randomisation, GPs completed a baseline survey based on a previous questionnaire administered by the research team [15,16] and others [22] to ascertain baseline knowledge and self-reported management of patients with atrial fibrillation.

### Recruitment of the patient cohort

**Figure 1:**
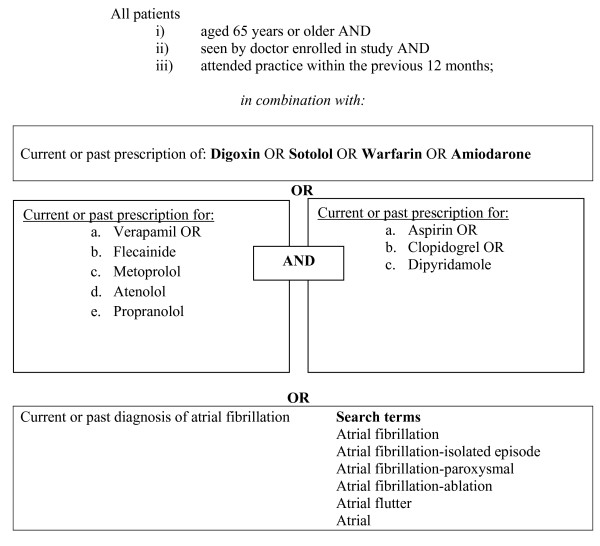
**Summary of electronic search strategy**.

The prevalence of atrial fibrillation is relatively low in patients over the age of 65 years [2,3]. As it was not feasible to search the records of all patients over the age of 65 years, a search strategy was applied to electronic prescribing records to identify patients before practices were randomised (Figure 1). The search strategy was limited to patients over the age of 65 years who had attended the practice within the last 12 months and had been issued prescriptions for medications commonly used to treat atrial fibrillation (Figure 1). This search strategy builds on work showing that selecting patients prescribed digoxin identifies patients with atrial fibrillation with high specificity (> 95%) [23,24]. In developing the search strategy, we piloted an earlier version in the practice of one GP not involved in the study and found that 85% of patients with a noted diagnosis of atrial fibrillation in their medical records were identified using medication search terms for current or past use of digoxin, amiodarone, sotalol, or warfarin.

Before randomisation, GPs or a member of the practice perused the list of patients meeting our age and medication search criteria and removed patients who had died, had a life expectancy of less than 12 months, or were affected by dementia or significant cognitive impairment. Patients with insufficient English language skills or no longer visiting the practice were also removed from the list.

An 'opt-out' consent process was approved by the administering institution's human research ethics committee. Patients meeting search and inclusion criteria were mailed a letter on GP and University letterhead explaining that their GP was involved in a research study and that researchers were requesting to review their medical record. Patients declining permission were advised to notify research staff by completing a form to return to the researchers via a business reply paid envelope or to notify research or practice staff of their decision via phone.

### General practitioner randomisation and allocation concealment

After patients had been contacted, GPs were randomised by a statistician external to the research project to ensure allocation concealment, into one of two groups: DESPATCH or a waiting-list control. All GPs sharing the same practice address (group practices) were randomised as one cluster and randomisation occurred on the same day for all GPs (October 13, 2009). GPs were first stratified by LGA. Within each stratum, they were then ranked by practice size (*i.e*., the number of patients contacted at baseline) before being randomly allocated into one of the two arms of the study using computer-generated random numbers. Block randomisation with a fixed block size of two was used to minimise the discrepancy in sample size at the individual level.

### The DESPATCH intervention

This is a multifaceted, tailored educational intervention comprising components designed to redress barriers to the translation of best evidence into clinical practice relevant to the management of NVAF. The DESPATCH intervention includes decisional support to improve confidence in decision making. The intervention was delivered within 12 months of randomisation.

### Academic detailing

**Figure 2:**
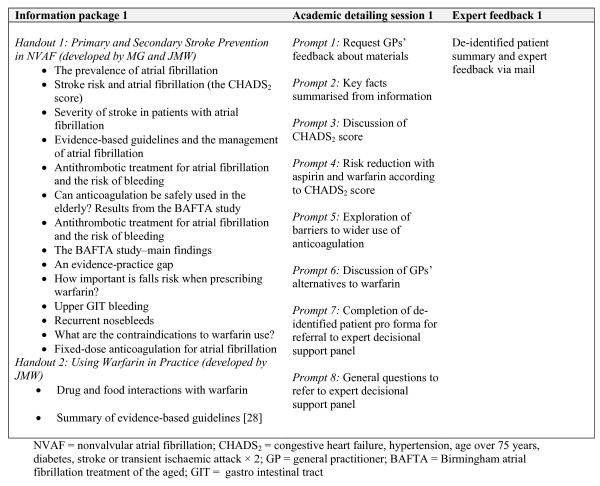
**Outline of DESPATCH intervention and its delivery: first phase**.

**Figure 3:**
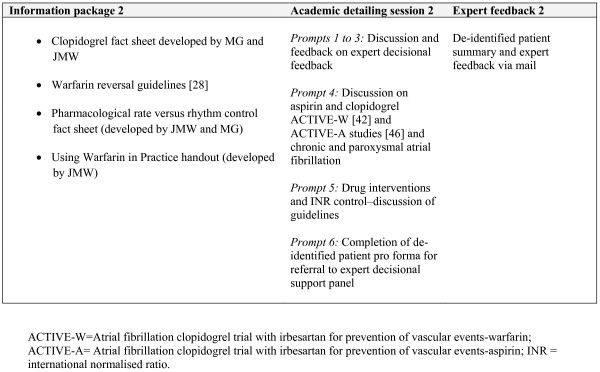
**Outline of DESPATCH intervention and its delivery: second phase**.

**Figure 4:**
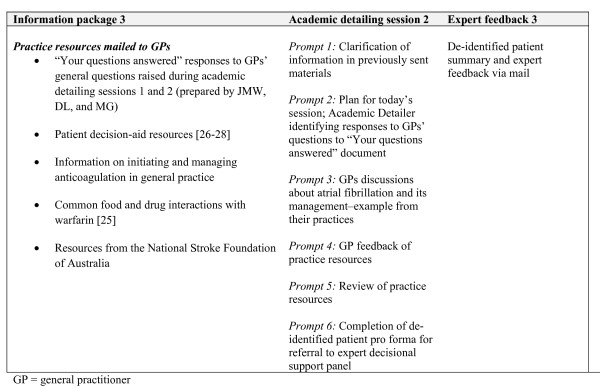
**Outline of DESPATCH intervention and its delivery: third phase**.

Medically trained peers were employed to deliver three academic detailing sessions via telephone. Prior to each of the three contacts, GPs received a mail out of resources from the research team (Figures 2,3,4). Resources included summaries of existing randomised controlled trials evaluating antithrombotic therapies, risk stratification using the CHADS2 score, information on common drug and food interactions with warfarin [25], and a patient decision aid adapted from an existing resource [26]. A patient question prompt sheet and a values-clarification exercise, modified from published resources [27,28], were included. All mailed materials were accompanied by a cover letter signed by JMW, DYL, NZ, and MG using electronic signatures.

Each academic detailing session comprised standardised prompts related to the mailed materials addressing barriers to the use of anticoagulation in their practice. During each academic detailing session, GPs were invited to identify a patient with atrial fibrillation about whose management they wish to receive specific feedback. The medical peers used a standardised pro forma for each GP-identified patient, requesting and recording information from GPs about patient medical history, stroke risk factors, current antithrombotic treatments, adverse events on antithrombotics, and any reasons for not prescribing anticoagulants. Academic detailers were instructed to calculate the CHADS2 score and provide evidence-based feedback using standardised information on antithrombotic treatment.

### Expert decisional support

After each academic detailing session, medical peers returned completed pro formas to the research team. On behalf of the GPs, the research team sought feedback from experts about the management of these patients. Experts comprised medical specialists in neurology and cardiology. Information from each de-identified completed pro forma was summarised onto one page. For each patient, a CHADS2 score and annual ischaemic stroke risk were reported. The one-page summary was emailed to specialists to provide written feedback, and this feedback was then mailed to GPs via the research team (Additional File 1).

During the development of our intervention, another team published a protocol for a cluster randomised controlled trial (cRCT) in primary care, evaluating a faxed one-page evidence-based statement referring to specific patients, with or without the signatures of local 'opinion leaders' [29]. The protocol provided a model for delivering this aspect of the intervention.

### Seminars

GPs in the intervention were invited to a workshop delivered by JMW. The workshop summarised current evidence on antithrombotic medication, risk stratification for patients with NVAF, and a discussion of barriers to the wider use of anticoagulation. Case studies were used to illustrate evidence-based patient management. The workshop was based on a published education module authored by JMW and MG for the National Stroke Foundation of Australia [30].

GPs randomised to the control group received evidence-based guidelines [31,32] by mail and were invited to a seminar delivered by DYL about a topic in cardiology unrelated to atrial fibrillation.

### Follow-up

We define our follow-up period as the 12-month interval following the date practices were randomised (October 13, 2009).

## Outcome assessment by medical record audit

In a blinded, independent medical record audit, research nurses will collect data to enable our assessment of the outcomes. Nurses will locate records of patients identified by the search strategy applied prior to randomisation, excluding records of patients who had refused permission. For cost and ethical reasons, auditing of posttest and baseline data is to be carried out after the intervention has been delivered. Auditing at posttest avoids the ethical dilemma of withholding feedback about suboptimal practice before completion of the study. Nurses carrying out data collection have not previously been involved with the project and will be employed through a company supplying contracted health services staff.

## Quality assurance of medical record audit

Depending on available resources, we aim to repeat the audit process in a random sample of between 5% to 10% of practices, obtaining estimates of inter- and intrarater reliability.

## Ascertainment of atrial fibrillation

Using a standardised audit form, the trained nurse auditors, blinded to the study design, aims, and group allocation, will apply a standardised checklist to first determine whether or not a patient has a recorded diagnosis of atrial fibrillation.

Any diagnosis of atrial fibrillation noted in the medical record, appearing in specified test results (electrocardiograms, Holter monitoring, and transesophageal or transthoracic echocardiograms), referral letters, specialist correspondence, or hospital discharge summaries, will identify patients as having atrial fibrillation. The date of the recorded entry, correspondence, or test result will be noted. In the absence of a diagnosis of atrial fibrillation, atrial flutter will be recorded where noted in the medical record.

For the purposes of distinguishing between cases of atrial fibrillation (or atrial flutter) first noted before or after randomisation, nurses are instructed to exhaust the standardised checklist and proceed through a set of instructions directing them to note if atrial fibrillation was diagnosed on or after a chosen date (October 1, 2009), approximating the date of randomisation on October 13, 2009. Patients whose diagnosis of atrial fibrillation was noted only after randomisation will be considered 'newly diagnosed' and excluded, minimising the possibility of biased patient selection postrandomisation. To maintain blinding of the study design and aims, nurses are informed that one of the project aims is to distinguish between newly diagnosed and established cases of atrial fibrillation (or atrial flutter) to determine the incidence of newly diagnosed cases over a period of 12 months. Nurses are instructed to collect data on all cases of atrial fibrillation (or atrial flutter), irrespective of when the diagnosis was noted.

When collecting information about comorbidities (see below), nurses will also record diagnoses of mitral stenosis, mixed mitral valve disease, rheumatic mitral valve disease, or mitral valve replacement, allowing the researchers to exclude these cases as examples of valvular atrial fibrillation. Cases of atrial flutter, in the absence of a recorded diagnosis of atrial fibrillation, will be regarded as equivalent to a diagnosis of atrial fibrillation in keeping with international evidence-based guidelines recommending identical management of these cardiac arrhythmias [5].

## Ascertainment of comorbidities and antithrombotic treatment

Comorbidities needed to calculate the CHADS2 score and other cerebrovascular risk factors will be noted. Nurses will search the medical record from January 1, 2004 onwards to allow ascertainment of medical history available at the time of randomisation.

Nurses will record antithrombotic treatments and dates of noted use. Dates and results of international normalised ratio (INR) testing and recorded suspensions of anticoagulation, if prescribed, are also noted. These data were collected to enable ascertainment of antithrombotic use current at randomisation and outcome assessment during the 12-month period after randomisation (see below).

## Cerebrovascular and bleeding events

All entries of cerebrovascular and bleeding events or episodes noted in the medical record from January 1, 2004 will be recorded so that the history of these events current at the time of randomisation and new events occurring during the 12 months after the date of randomisation will be recorded (see below).

## Demographic characteristics

Sex and year of birth will be documented.

## Primary outcome

We have defined the primary outcome as the proportion of patients with atrial fibrillation over the age of 65 years noted to be on treatment with oral anticoagulation at two prespecified time periods: (1) at *any *time in the 12 months from the date of randomisation and (2) currently (as defined below). Medical record notations of oral anticoagulant treatment, including prescriptions, consultation notes, referral letters, correspondence from specialists, hospital discharge summaries, and dates of INR results (with INR levels greater than 1.2), will be considered to be an indication that the patient is receiving anticoagulation on the date of the notation.

Anticoagulation indicated in the last three months of the 12-month period from randomisation will be considered 'current' if there is no noted suspension of warfarin or if a suspension is noted to be temporary. Three months is chosen as current to ensure adequate ascertainment of our anticoagulation indicators, such as doctor's follow-up notations, prescriptions, and correspondence.

At the time the study was devised and at the time of writing, warfarin was the only locally approved anticoagulant for the management of atrial fibrillation. During the study period, two fixed-dose oral anticoagulants (dabigatran and rivaroxaban) [33,34] had been under investigation in randomised controlled trials. Therefore, in addition to warfarin, these two treatments will also be considered as oral anticoagulant treatment in the unlikely event that patients in this project are receiving the drugs in trials or are receiving these treatments off-label. Other types of anticoagulation, such as clexane (enoxaparin) and heparin, will not be considered in our primary or secondary outcome assessment. These medications are not administered orally and are not usually indicated for long-term anticoagulation in NVAF.

## Secondary outcomes

### The proportion of patients prescribed antithrombotic treatment judged as 'appropriate' according to stroke risk

Stroke risk will be assessed using a validated, evidence-based risk stratification scheme and evidence-based guidelines. At the time the study was initiated, the evidence-based risk stratification scheme often endorsed by national and international guidelines was the CHADS2 score [35]. Local guidelines recommend anticoagulation with warfarin for patients with a CHADS2 score of 2 or higher, aspirin or warfarin for patients with a CHADS2 score of 1, and aspirin for patients with a CHADS2 score of 0. This outcome will be measured for *any *time within 12 months of postrandomisation and for current use, as defined above. Only comorbidities noted in the medical record prior to the date of randomisation will be considered in the calculation of the CHADS2 score.

### The proportion of patients prescribed antithrombotic treatment judged as appropriate as above, incorporating quality control criteria for anticoagulation use

If patients are receiving anticoagulation with warfarin, antithrombotic treatment will be judged to be appropriate only where patients receive warfarin according to the above criteria for appropriate antithrombotic treatment *and *where quality control of warfarin is adequate. Adapting a definition devised by McAlister *et al*. [24], quality control of warfarin will be considered adequate if INR levels are measured at least monthly, from the first date patients are known to be taking warfarin during the 12-month follow-up period, and if at least 67% of INR levels are between 2.0 and 3.0. A minimum of monthly INR measurements are recommended in local guidelines [32] and 67% of INR readings within the therapeutic range has been achieved in randomised evaluations of warfarin [6,8]. We will not impose any minimum required number of INR results to calculate this outcome. In the instance of patients receiving new fixed-dose anticoagulants, quality control criteria will be assumed to be met without INR evaluation, as these medications do not require monitoring.

### The percentage of time patients used oral anticoagulation over the 12-month postrandomisation follow-up period

The first recorded date of oral anticoagulation noted in the medical record during the 12-month follow-up period will be considered the index date. We will assume anticoagulation use recorded up to three months before randomisation will indicate anticoagulation was used on the date of randomisation (*i.e*., start of the follow-up period), provided treatment had not been suspended. For these patients, the index date will be assumed to be the date of randomisation.

Each noted suspension of oral anticoagulation and the dates of and reasons for suspension will be recorded during the 12-month follow-up period. The number of days from the index date until treatment is suspended will be calculated. The next noted date of oral anticoagulation use will indicate that treatment was reinstated. Noted use of anticoagulation in the medical record at least once every three months from the index date or date of reinstatement will be assumed to indicate continuous treatment throughout that three-month period in the absence of any recorded suspension of treatment.

Where there are no subsequent dates recording oral anticoagulation use beyond a three-month period, we will assume treatment was suspended three months from the last date recording oral anticoagulation use. Patients without a recorded note of oral anticoagulation will be considered to have had zero days of use.

The number of days of oral anticoagulation use will be summed and divided by the denominator (*i.e*., 365 days) to calculate the percentage of days of anticoagulation use.

### Adverse events comprising the following individual outcomes: (a) the proportion of patients with systemic embolism or 'total stroke', (b) major bleeding, (c) minor bleeding, and (d) any bleeding event recorded during the 12-month follow-up period

(a) Systemic embolism and total stroke rates have been used in studies evaluating antithrombotic medications. Applying standard definitions [6,8], we consider total stroke as comprising ischaemic stroke and haemorrhagic cerebral events (intracerebral, intracranial and subarachnoid haemorrhages, and subdural haematoma) and stroke not otherwise specified as either haemorrhagic or ischaemic. Haemorrhagic stroke will include traumatic and nontraumatic intracranial haemorrhage, subarachnoid haemorrhage, and subdural haematoma. This is in keeping with definitions applied in previous trials and systematic reviews of randomised controlled trials of antithrombotic treatment in atrial fibrillation [6,8]. We will also consider systemic embolism, haemorrhagic stroke, ischaemic stroke, and stroke not otherwise specified as four individual outcomes. TIA will comprise a separate cerebrovascular outcome and not be included in the composite outcome, in keeping with previous definitions of total stroke [6,8]. The likely rarity of these events, however, may preclude robust statistical analysis for these individual outcomes.

(b) A major bleeding event is defined as a haemorrhagic stroke or other bleeding associated with a hospital admission or blood transfusion. All other bleeding events will be classified as (c) minor bleeding, including anaemia and bruising. Our classifications of major and minor bleeding correspond with definitions used elsewhere [6,8]. We will also compare groups on the occurrence of (d) *any *bleeding event, in recognition that this outcome will likely involve a greater number of events, permitting a more robust statistical analysis and also minimising the effect that misclassification will have on our definitions of major and minor bleeding events.

### Subgroup analyses

For our primary outcome, we will carry out subgroup analyses, testing for interaction effects between four variables and trial arm status: (1) CHADS2 scores, (2) patient age (65 to 74 years, 75 to 84 years, and 85+ years), (3) recorded use of oral anticoagulation current at time of randomisation (that is, within three months of randomisation; yes or no), and (4) patient sex.

### Baseline comparisons between DESPATCH intervention and control groups

We will compare groups on key patient and practice characteristics current on the date of randomisation. Specifically, we will compare the DESPATCH and control groups on the numbers of patients identified with atrial fibrillation, patient sex, age (mean and median differences), CHADS2 scores (0, 1, 2+, and mean scores), use of oral anticoagulation current at time of randomisation (that is, up to three months before randomisation; yes or no), and whether patients were recruited from practices where one or more than one GP participated in the study.

### Losses to follow-up

Patients identified as having atrial fibrillation without any recorded contact with GPs during the follow-up period will be considered lost to follow-up. The proportion of patients lost to follow-up or with partly completed follow-up will be compared across groups.

### Sample size

Our sample size estimate was powered to detect a clinically important difference between groups for our primary outcome. We considered a 10% difference in the primary outcome to be clinically important. We assumed that 50% of patients with atrial fibrillation managed by GPs assigned to the control arm received anticoagulation; assuming a 50% use of anticoagulation in the control arm will produce a conservative (*i.e*., larger) sample size estimate. To detect a difference of 10% (*e.g*., 60% vs. 50%) in the primary outcome between intervention and control groups, with 80% power at the 5% level of significance, we would require 407 eligible patients per group in a trial in which the unit of randomisation is the patient [36].

As practices (clusters) were randomised, we needed to allow for the correlation between the outcomes of patients from the same cluster. We have inflated the sample-size estimate by the design effect (DEFF); DEFF = [1 + (*m *- 1)ρ], where *m *is the average number of patients per cluster and ρ is the intracluster correlation coefficient (ICC) that quantifies the amount of within-cluster correlation for the outcome of interest [37]. An estimate for the ICC for warfarin uptake in patients with atrial fibrillation recruited within general practice clusters is 0.029 [11]. We have conservatively chosen an ICC of 0.04 in recognition of the imprecision with which ρ is estimated. We estimate an average of 20 eligible patients with NVAF per GP will be identified. This estimate assumes an average of 350 eligible patients over the age of 65 per GP, a prevalence rate for NVAF in general practice of ~8.6% [38], that our computerised search strategy will identify 85% of patients with atrial fibrillation, and that 80% of patients will not refuse permission for an independent medical record audit. Based on previous experience of recruiting GPs [39], we expect to recruit an average of 1.25 GPs per practice, yielding an average sample size of 25 patients per practice (1.25 × 20). The estimated DEFF is 1.96 (*i.e*., [1 + (25 - 1)0.04]). Therefore, we require 798 patients per group (*i.e*., 407 × 1.96). Assuming 10% will die, move away during the study, or be lost to follow-up, our revised sample size is 887 per group. We therefore aimed to recruit 36 practices per group (*i.e*., 887 ÷ 25 patients per practice), 72 practices in total, and 90 GPs in total (72 × 1.25 GPs per practice). To further allow for 10% drop out at practice level, we aimed to recruit 40 practices per group (80 in total, or 100 GPs), yielding a total patient sample of 2,000 (100 GPs × 20 patients or 80 practices × 25 patients) or 1,000 per group.

### Statistical analysis

All outcomes will be analysed according to the intention-to-treat principle, where patients are analysed according to the arm to which their practice cluster was allocated. Analyses of the outcomes will be implemented using marginal logistic regression models using generalised estimating equations (GEEs), with information sandwich ('robust) estimates of standard error to allow for within-cluster (within-general practice) correlation for dichotomous outcomes [40]. An exchangeable correlation structure will be specified for the marginal models using GEEs. Clustering will be accounted for only at the practice level as this is the unit of randomisation. Analyses will adjust for stratification of GPs by LGA.

In addition to unadjusted analyses, we will carry out adjusted analyses to allow for the effect of characteristics, current at the time of randomisation, as noted above.

Percentage of inter- and intrarater agreement and kappa coefficients will be calculated to quantify rates of reliability for the collected audit data [41].

Significance of the study results, specifically, the effect of the intervention on primary and secondary outcomes, will be ascertained by examining the magnitude of the estimated effect of the intervention and corresponding 95% CIs. When testing for interaction terms, a *p *value of < .05 will be used to determine significance of the effect. Analyses will be carried out blinded to group allocation.

### Warfarin use in antithrombotic combinations

As with aspirin, warfarin may also be used in combination with other antithrombotics, particularly aspirin and clopidogrel. In patients with atrial fibrillation, double or triple therapy using aspirin and/or clopidogrel with warfarin may be used for varying periods in the context of acute coronary artery disease and particularly in the context of recent coronary artery stenting [42-45]. Safety and efficacy of double or triple antithrombotic treatment have not been specifically evaluated in the context of NVAF [45], and these treatment choices are likely to be made by specialists. General-practice medical records may not contain sufficient documentation of the considerations used in these decisions. Where warfarin is an appropriate choice in NVAF, patients receiving warfarin with aspirin and/or clopidogrel will be considered to fulfill the primary outcome criteria.

### Sensitivity analyses of antiplatelet medications

Other antithrombotics, namely clopidogrel or slow-release dipyridamole, may be used in lieu of aspirin or in addition to aspirin. We expect to encounter a sizable proportion of patients on clopidogrel, dipyridamole, and combinations. Existing national guidelines do not recommend clopidogrel over aspirin for stroke prophylaxis in atrial fibrillation, and clopidogrel is not an evidence-based substitute for warfarin [42-45]. Clopidogrel may, however, be considered appropriate for patients with an intolerance or allergy to aspirin and in settings of acute coronary syndromes and coronary artery stenting, where it is often used in combination with aspirin [36-39]. We expect it will be difficult to ascertain reasons for clopidogrel use where aspirin alone is considered the appropriate evidence-based choice in NVAF. We will carry out sensitivity analyses for our secondary outcome of appropriate antithrombotic use also accepting clopidogrel, aspirin and clopidogrel, dipyridamole, and aspirin and dipyridamole as appropriate, where aspirin would be the evidence-based choice.

### Sensitivity analyses assessing the effect of losses to follow-up on study outcomes

In order to test whether our results for the main outcome is robust against exclusion of patients lost to follow-up, we will rerun analyses for our primary outcome excluding patients with missing data and assuming patients with missing follow-up are not receiving anticoagulation.

### Quality assurance of data entry and data management

All data will be entered by two individuals blinded to group allocation. Data cleaning will occur blinded to group allocation. Once data are cleaned, the database will be locked down; the statistician responsible for allocation will then provide the study statistician with codes for group allocation to enable blinded analysis.

### Ethical considerations

For ethical reasons, GPs will receive summaries of data extracted from medical records for all their patients recruited into the study. GPs allocated to the control group will receive all project materials and access to specialist decisional support for a period of time up to that which was available to GPs allocated to receive DESPATCH.

The study has ethical approval from the institutional ethics committee of the administering institution, The University of New South Wales (UNSW HREC Reference Number 07068).

## Discussion

Improved uptake of antithrombotics in patients with NVAF will reduce stroke risk, death, and disability. Peak national and international medical authorities have recommended increased uptake of appropriate antithrombotics for patients with NVAF. If successful, DESPATCH will generate high-quality evidence supporting a strategy to close an evidence-practice gap, reducing stroke risk in patients with NVAF.

One potential challenge common to studies evaluating clinician behaviour change is the possibility that the control arm becomes 'contaminated' because of secular trends in clinical practice that cannot be anticipated or controlled. Arguably, clinician behaviour is unlikely to have shifted appreciably during our study period, as doctors' concerns about warfarin risk appear entrenched, despite favourable evidence from almost 20 published trials over the past 15 years.

The DESPATCH intervention specifically targets GP attitudes and concerns about anticoagulation, aiming to redress knowledge gaps and increase their confidence when using anticoagulation. The intervention provides tailored advice and decisional support from experts in stroke prevention, neurology, and cardiology.

This intervention has been delivered and data collection has commenced and is expected to be completed in the second half of 2011.

## Competing interests

JG is on the Editorial Board of *Implementation Science*. No other conflicts are declared by the authors.

## Authors' contributions

JMW initiated the group's interest in developing an intervention in general practice to reduce stroke risk in patients with atrial fibrillation. MG, JMW, and NZ conceived the study; all authors contributed to the protocol design and submission for funding and are named Chief Investigators on the funded proposal. MG is the lead investigator on the study. OCU provided substantial input to the statistical analysis and sample-size calculation; MG and JMW designed the intervention, JMW prepared authored information resources, and MG wrote the first draft with substantial input and revision by JMW. JMW, DYL, and MG defined the outcomes for the study. All authors reviewed and provided feedback on the protocol. All authors have read and approved the final submission.

## Supplementary Material

Additional file 1 **Example of specialist, expert feedback about a GP identified case**. Click here for file
