# Supplementary material for: Study protocol: the DESPATCH study: Delivering stroke prevention for patients with atrial fibrillation - a cluster randomised controlled trial in primary healthcare
Source: Implement Sci. 2011 May 20;6:48. doi: 10.1186/1748-5908-6-48 (PMC3121604; doi:10.1186/1748-5908-6-48)
Supplement: Additional file 1 — Example of specialist, expert feedback about a GP identified case. [file 1748-5908-6-48-S1.DOC]

Additional File 1: Example of specialist, expert feedback about a GP identified case*

ID: [XXXX]

**80 year old [female/male] patient with atrial fibrillation (non-valvular) without thyrotoxicosis**

SUMMARY OF PATIENT HISTORY

| **Type of AF** | [Chronic/Paroxysmal] |
| --- | --- |
| **Ischaemic stroke risk factors** | Congestive Heart Failure  Previous Stroke |
| **CHADS_2_ Score** | 4 Annual Ischaemic Stroke Risk: 8.5% |
| **Other relevant co-morbidities** | A falls history with “high falls risk”  Past subdural haemorrhage while on Warfarin |
| **Antithrombotic medications** | Aspirin |
| **Other medications** | Beta-blockers  Paracetamol PRN |
| **Anticoagulant use -Current** | No  Patient has previously used warfarin which was discontinued due to history of a subdural haemorrhage occurring almost one year ago. |
| **Adverse events/concerns**  **whilst on warfarin** | Intracranial or intracerebral haemorrhage (including subdural haemorrhage and subarachnoid haemorrhage). |
| **Reasons for not**  **prescribing warfarin** | Due to past history of subdural haemorrhage while on Warfarin |
| **Home Medicines Review (HMR) considered?** | No |
| **Webster packaging considered?** | No |
| **GP questions re management/comments** | Is the patient's past subdural haemorrhage a contraindication? |
|  |  |

**Specialist comments:** Annual Ischaemic Stroke Risk: 8.5%. Previous subdural haematoma is not a contraindication to warfarin. Risk-benefit studies have found that despite risks associated with warfarin, the benefits outweigh the risks even in patients who fall. Hence, warfarin is still preferred to aspirin or no therapy in elderly patients at risk of falls. Options to consider include arranging aged care assessment or alternatively a physiotherapist and/or occupational therapist to assess patient’s gait and/or gait assistance devices as well as patient’s home environment to see if there are further ways to reduce their risk of falls and/or falls-related injury.

#2 Spontaneous subdurals with acute symptoms are a difficulty for all antithrombotics. However, falls/trauma related SDH is less of an issue. This patient has a very high stroke risk estimated from a CHADS2 to be 8.5% per year and may be higher still in frailer patients with a falls risk. Their greatest risk is likely to be an AF-related stroke which confers a 20% risk of early mortality (and about 40% at one year) and a 60% risk of disability. It is estimated that a patient has to fall over 295 times for the increased risk of harm to overcome the risk of benefit from warfarin (Gage et al, American Journal of Medicine, 2005: 118: 612-7; Man-Son-Hing et al; Archives of Internal Medicine;1999; 159: 677-865.) Aspirin is the only evidence-based alternative to warfarin but loses effectiveness with increasing age and the absolute benefit of warfarin increases with age. The recommended INR would still be 2-3 with a specific target of 2.5, as used in BAFTA.

**Note: This example is based on an actual patient although demographic and medical information has been changed so that these details do not correspond to a real case.*
